# From empirical practice to precision medicine: developing a methodology for adjusting traditional Chinese medicine formula dosages based on eight multicenter clinical studies

**DOI:** 10.3389/fphar.2026.1815285

**Published:** 2026-06-29

**Authors:** Shanshan Tang, Hangyu Ji, Yashan Cui, Ying Zhang, Xuedong An, De Jin, Fengmei Lian

**Affiliations:** 1 Guang’an Men Hospital of China Academy of Chinese Medical Sciences, Beijing, China; 2 College of Traditional Chinese Medicine, Changchun University of Traditional Chinese Medicine, Changchun, China; 3 Xiyuan Hospital of China Academy of Chinese Medical Sciences, Beijing, China; 4 School of Traditional Chinese Medicine, Beijing University of Chinese Medicine, Beijing, China; 5 Hangzhou Municipal Hospital of Traditional Chinese Medicine, Hangzhou, China

**Keywords:** evaluation methods, research strategy, SMART design, symptom-driven dose adjustment, traditional Chinese medicine formulae

## Abstract

**Background:**

Modern medicine enables dose optimization based on pharmacokinetics-pharmacodynamics (PK-PD) models. In contrast, dose adjustment for traditional Chinese medicine (TCM) formulas lacks quantitative standards and relies largely on empirical practice. This is especially problematic in managing chronic diseases like type 2 diabetes, which require long-term dose titration. Key research bottlenecks include unclear dose-response relationships, a lack of sensitive biomarkers, and the absence of quantitative guidance for dynamic adjustment, hampering scientific decisions on 'when and how much to adjust.' Consequently, developing a scientific methodology for TCM formula dose adjustment is a crucial unmet clinical need.

**Methods:**

We conducted a two-phase study. Phase I (retrospective analysis) pooled patient data from seven multicenter clinical studies. Using multivariate and logistic regression alongside ROC analysis, we screened baseline and on-treatment variables to identify those most predictive of HbA1c reduction. This analysis established the optimal timing (week 4) and threshold (FPG reduction <0.5 mmol/L) for dose adjustment and modeled the magnitude of adjustment required. Phase II was a prospective, randomized, controlled trial that compared the efficacy and safety of a fixed-dose regimen versus a dynamic, response-guided titration strategy (the 'symptom-driven dose adjustment' protocol) based on the rules derived from Phase I.

**Results:**

The key findings of this study can be summarized as follows: a) A reduction in fasting plasma glucose (FPG) of <0.5 mmol/L at 4 weeks of treatment was established as the critical threshold for triggering a dose adjustment. b) Different TCM formulas (e.g., Gegen Qinlian Decoction (GQD) and Tang-min-ling pills (TM81) exhibited distinct dose-response relationships, indicating that the magnitude of dose adjustment should be determined on an individualized, formula-specific basis. c) Analysis of the dose-ranging clinical data for GQD indicated that a dose increase of approximately 50% is appropriate when the aforementioned indicator threshold is not met. d) The prospective validation study demonstrated that the symptom-driven dose adjustment group achieved superior outcomes in HbA1c, FPG, and other glycemic parameters compared to the fixed-dose group (P < 0.05).

**Conclusion:**

The 'symptom-driven dose adjustment' strategy provides a scientific basis for dosing traditional Chinese medicine formulas, enhances clinical efficacy, and facilitates the optimization of treatment regimens.

## 1 Introduction

The selection and dynamic adjustment of initial drug doses present a major challenge in the management of chronic diseases. Modern medicine, based on pharmacokinetic-pharmacodynamic (PK-PD) models, has clarified the dose-response relationships and therapeutic thresholds of drugs, allowing for precise dose titration in therapeutic regimens (Grant et al., 2023; Barrow and Lindsley, 2023). In contrast, the clinical application of Chinese herbal formulas still relies primarily on empirical dose adjustments, lacking quantitative standards. This leads to considerable disagreement on key questions such as ‘When should the dose be adjusted?’ and ‘By how much should it be adjusted?’—issues that are often addressed through experiential practice, resulting in a black-box approach. This limitation is particularly pronounced in the long-term management of chronic diseases, which requires dynamic dose titration, and ultimately compromises the consistency and reproducibility of the therapeutic effects of Chinese herbal formulas.

Currently, dose optimization of traditional Chinese medicine (TCM) compound formulas faces three core challenges: (1) The complex composition of herbal formulas results in unclear dose-response relationships; (2) There is a lack of sensitive biomarkers to guide dosage adjustment, leading to a deficiency in objective evidence for precise dose titration; (3) The concept of symptom/syndrome-driven dose adjustment (Lian, 2011) remains largely empirical, lacking robust evidence-based support. Existing strategies, such as ‘increasing the dose if ineffective’ or ‘reducing the dose once the condition improves,’ are overly broad and provide insufficient guidance for treating specific diseases (i.e., when to adjust the dose and by how much). Although modern clinical trial designs like randomized controlled trials (RCTs) have been increasingly adopted in TCM research in recent years, most studies still employ fixed-dose protocols and endpoint evaluation models, failing to reflect the characteristic TCM treatment principles of ‘syndrome differentiation and treatment’ and ‘dynamic adjustment.’ Therefore, constructing a scientific and quantifiable strategy for adjusting the dosage of TCM compound formulas has become a critical issue that urgently needs to be addressed.

In this study, we used Chinese herbal formulas for the treatment of type 2 diabetes mellitus (T2DM) as a paradigm and adopted a combined retrospective and prospective study design. First, we screened early efficacy predictors and determined the dose-adjustment threshold based on large-scale retrospective data. Second, we analyzed the dose-response relationships of different formulas to illustrate the necessity of individualized dose adjustment. Third, we quantified the specific dose-increment magnitude using multi-dose trial data. Finally, we validated the effectiveness of this strategy through a prospective randomized controlled trial. This study is the first to establish a comprehensive dose adjustment system comprising a clear time window (4 weeks), an objective biomarker (change in FPG), and a quantifiable dose adjustment magnitude (increase by half-dose). It is particularly noteworthy that, upon completion of the research, we found this strategy to be highly aligned with the core concept of modern adaptive clinical trial designs (Sequential Multiple Assignment Randomized Trial, SMART) (Bigirumurame et al., 2022; Kidwell and Almirall, 2023), while simultaneously retaining characteristics of traditional Chinese medicine: achieving personalized therapy through precise dose titration of a single formula (rather than protocol switching). This finding provides a new paradigm for research on Chinese herbal formulas. This study facilitates a shift in T2DM herbal medication dosing from empirical judgment to data-driven decision-making. The established dose adjustment system demonstrates good clinical operability and potential for broad application. It can be extended to research on other herbal formulas in the future, promoting the advancement of precision medicine in traditional Chinese medicine.

## 2 Materials and methods

The eight studies referenced in this article were conducted at over ten hospitals, including Guang’anmen Hospital, Peking University Jishuitan Hospital, China-Japan Friendship Hospital, and Dongzhimen Hospital affiliated with Beijing University of Chinese Medicine. All eight studies were approved by ethics committees, and all participants signed informed consent forms prior to study commencement. The research was conducted in accordance with the principles of the Declaration of Helsinki.

### 2.1 Study design

#### 2.1.1 Retrospective study: reanalysis of retrospective multicenter data

##### 2.1.1.1 Study design and participants

This study pooled seven previously published multicenter, randomized, parallel-group clinical trials (Xu et al., 2015; Kang et al., 2024; Yu et al., 2018; Tong et al., 2013; Tong et al., 2009; Lian et al., 2008) conducted by the research team. In all studies, TCM formulas were used alone to treat type 2 diabetes mellitus (T2DM), with a treatment duration of ≥12 weeks and glycated hemoglobin (HbA1c) as the primary efficacy endpoint. The included patients were treatment-naive or had not previously received standardized treatment. Patients taking Western hypoglycemic agents or other TCM products, as well as those with severe complications, were excluded. To ensure the statistical power of the regression and ROC analyses, this retrospective analysis required a minimum sample size of 1,200 participants; a total of 1,809 cases (with 1,127 variables) were ultimately included, fully meeting the analytical requirements. All original studies were ethically approved, and patients provided written informed consent (see Table 1 for details).

**TABLE 1:** Summary of key characteristics of seven studies in study 1.

Database name	Research drug name	Research design type	Sample size	Grouping/Personnel assignment
Database 1	Jiangtang Tiaozhi formula (JTTZF)	Multicenter, randomized, active-controlled, open-label clinical trial	450	Experimental group, metformin group (1:1)
Database 2	GQD (Kudzu root as the principal herb)	Randomized, double-blind, dose-parallel, multicenter clinical trial	120	High, medium, and low dose groups (1:1:1)
Database 3	GQD (Coptis as the primary herb)	Randomized, double-blind, dose-parallel, multicenter clinical trial	120	High, medium, and low dose groups (1:1:1)
Database 4	GQD	Randomized, double-blind, placebo-controlled clinical trial	224	High-, medium-, and low-dose groups, placebo group (1:1:1:1)
Database 5	Qingre Jiangzhuo prescription	Randomized, positive-control, open-label clinical trial	205	Experimental group, metformin group (1:1)
Database 6	TM81	Multicenter, randomized, double-blind, placebo-controlled clinical trial	480	Experimental group, placebo group (3:1)
Database 7	TM81	Randomized, double-blind, parallel-group, multicenter clinical trial design	210	High-dose group, medium-dose group, placebo group (1:1:1)

Database 1: JTTZF, treatment for T2DM, with obesity and hyperlipidemia.

Database 2: Clinical Study on Dose-Response Relationship of GQD, Decoction in Treating T2DM (Pueraria Root as Primary Herb).

Database 3: Clinical Study on Dose-Response Relationship of GQD, in Treating T2DM (Coptis as Primary Herb).

Database 4: Clinical Study on the Dose-Response Relationship of GQD, Treatment for T2DM (GQD, Formula).

Database 5: Randomized, Parallel-Controlled, Multicenter Clinical Study of Qingre Jiangzhuo prescription and Metformin for the Treatment of T2DM.

Database 6: Multicenter, Randomized, Double-Blind, Parallel-Group Phase III, Clinical Trial Comparing TM81 with Placebo for the Treatment of T2DM.

Database 7: TM81 versus Placebo in a Multicenter, Randomized, Double-Blind, Parallel-Group Phase II, Clinical Trial for the Treatment of Type 2 Diabetes Mellitus.

##### 2.1.1.2 Data inclusion and statistical analysis

Data harmonization and standardization were conducted in accordance with the provisions of the research protocol: single-ingredient drugs were treated as individual units, while compound formulations were broken down by individual ingredients; drug names were standardized in accordance with the Chinese Pharmacopoeia; symptom names were standardized in accordance with national industry standards; drug dosages were uniformly expressed in “g”; and all clinical indicators were converted using internationally accepted units. The clinical research database was established using EpiData software. Existing data were imported or independently double-entered by two researchers. After data verification, an analysis database was formed, ultimately integrating data from 1,809 patients across 7 clinical studies (including 1,127 variables).

Quantitative data are presented as mean ± standard deviation. Statistical analysis followed a three-step process: (1) Multivariate regression and binary logistic regression were applied to analyze the correlation between changes in indicators at various time points relative to baseline and changes in HbA1c; (2) Receiver operating characteristic (ROC) analysis was performed on the selected indicators, and the area under the curve (AUC) was calculated to assess their predictive performance for ΔHbA1c; (3) For indicators demonstrating significant predictive performance, the chi-square test combined with the Youden index maximization principle was used to determine the optimal cutoff values for distinguishing changes in treatment efficacy.

Data analysis strategy: Based on the aforementioned database and analytical methods, the retrospective data analysis in this study was conducted in the following sequence: First, using data from all seven studies, we completed indicator screening and threshold determination; second, we selected two studies with multi-dose designs (GQD and TM81) from the seven studies and analyzed their dose-response relationships separately to demonstrate that the dosage adjustment range must be determined individually for different compound formulas; finally, the multi-dose trial of GQD—which features the most complete data and the clearest dose gradient—was selected to calculate specific dosage adjustment ranges (as a case demonstration). The results of the above steps will be reported sequentially in the Results section.

##### 2.1.1.3 Study results

The research team incorporated and analyzed data from 1809 cases across seven previously completed clinical studies to establish core indicators and timing for dose adjustment guidance. Through data analysis of multiple-dose clinical trials, the magnitude coefficient for dose adjustment was estimated.

#### 2.1.2 Determination of core indicators for guiding dose adjustment

Through systematic analysis of multiple indicators—including demographic characteristics, vital signs, and laboratory parameters—at different assessment time points relative to baseline changes and their relationship with final glycated hemoglobin variation (ΔHbA1c), it was found that fasting plasma glucose (FPG) variation serves as a key sensitive indicator for predicting ΔHbA1c. Preliminary screening via multivariate regression analysis revealed that FPG changes at multiple time points (weeks 4, 6, 8, 10, and 12) were significantly correlated with ΔHbA1c (P < 0.05). Based on the above analysis, FPG was selected as the core indicator to guide dose adjustment in the ‘symptom-driven dose adjustment' strategy (see Table 2).

**TABLE 2:** Correlation analysis between changes in indicators at different time points and changes in HbA1c.

Timing	Indicators (relative change from baseline at each time point)	β	OR	*P*-value	95% CI
4 weeks	Weight	−0.303	0.739	0.152	(0.489,1.117)
	BMI	0.732	2.080	0.208	(0.666,6.499)
	Waist circumference	0.080	1.083	0.528	(0.845,1.390)
	Hip circumference	−0.105	0.900	0.375	(0.714,1.135)
	Waist-to-hip ratio	−10.948	<0.001	0.395	-
	SBP	<0.001	1.000	1.000	(0.985,1.015)
	DBP	0.033	1.034	0.002	(1.012,1.055)
	Heart rate	−0.010	0.990	0.337	(0.970,1.011)
	FBG	−0.245	0.783	<0.001	(0.730,0.840)
	2hBG	0.007	1.007	0.739	(0.968, 1.047)
6 weeks	FBG	−0.245	0.783	<0.001	(0.732,0.837)
	2hBG	0.008	1.008	0.673	(0.971,1.047)
8 weeks	Weight	−0.326	0.722	0.080	(0.501,1.039)
	BMI	0.795	2.214	0.122	(0.808,6.064)
	Waist circumference	0.069	1.072	0.520	(0.868,1.324)
	Hip circumference	−0.106	0.899	0.292	(0.738,1.095)
	Waist-to-hip ratio	−11.502	<0.001	0.293	-
	SBP	−0.013	0.987	0.100	(0.971,1.003)
	DBP	0.037	1.037	<0.001	(1.016,1.059)
	Heart rate	−0.004	0.996	0.759	(0.974,1.020)
	FBG	−0.314	0.730	<0.001	(0.682,0.782)
	2hBG	0.032	1.033	0.076	(0.997,1.070)
10 weeks	FBG	−0.300	0.741	<0.001	(0.693,0.792)
	2hBG	0.019	1.019	0.271	(0.985,1.054)
12 weeks	Weight	−0.349	0.705	0.011	(0.539,0.922)
	BMI	0.869	2.385	0.021	(1.142,04.982)
	Waist circumference	0.205	1.228	0.094	(0.965,1.562)
	Hip circumference	−0.220	0.803	0.053	(0.643,1.003)
	Waist-to-hip ratio	−24.775	<0.001	0.046	(<0.001,0.622)
	SBP	<0.001	1.000	0.935	(0.989,1.012)
	DBP	0.016	1.016	0.035	(1.001,1.032)
	Heart rate	0.007	1.007	0.249	(0.995,1.020)
	FPG	−0.137	0.872	<0.001	(0.818,0.929)
	2hPG	−0.059	0.943	0.001	(0.912,0.975)
	Insulin	−0.002	0.998	0.765	(0.987,1.319)
	TC	0.112	1.119	0.180	(0.949,1.062)
	TG	−0.012	0.988	0.732	(0.921,1.060)
	HDL	0.002	1.002	0.993	(0.646,1.555)
	LDL	−0.091	0.913	0.392	(0.741,1.124)

#### 2.1.3 Determining the timing for dose adjustment

Through predictive efficacy analysis of fasting plasma glucose change values (ΔFPG) at multiple time points (weeks 4, 6, 8, and 10), combined with clinical practice, prolonged dose adjustments may delay patient management. Therefore, ΔFPG at week 4 was determined as the optimal indicator for dose adjustment. At this optimal time point (Week 4), maximization analysis of the Youden index revealed ΔFPG <0.5 mmol/L as the optimal cutoff threshold (Youden index = 12.08%; sensitivity 49.02%, specificity 63.06%). The overall discriminatory performance of this threshold significantly exceeded that of other thresholds at week 4. In summary, ΔFPG <0.5 mmol/L at week 4 was definitively established as the objective threshold for triggering dose adjustment.

#### 2.1.4 Establishing the basis for dose adjustment calculations

To establish a quantitative basis for dose adjustment of TCM formulas, this study analyzed the dose-response relationships using data from two clinical trials with multi-dose designs: GQD and TM81. The results revealed distinct dose-response profiles between the two formulas. For TM81, a significant negative linear correlation was observed between dose and the 12-week reduction in glycated hemoglobin (ΔHbA1c) (β = −0.069, P < 0.001). In contrast, GQD did not demonstrate a significant linear dose-response relationship (β = −0.023, P = 0.066), suggesting that its therapeutic effect may reach a plateau within the studied dose range (see Table 3).

**TABLE 3:** Linear regression results for 12-week HbA1c efficacy at different doses.

Measure	β	*P value*	Lower limit of 95% CI for β	Upper limit of 95% CI for β
GQD	​	​	​	​
Intercept	−0.408	<0.001	−0.627	−0.189
Dose	−0.023	0.066	−0.048	0.002
TM81	​	​	​	​
Intercept	−0.429	0.002	−0.700	−0.158
Dose	−0.069	<0.001	−0.104	−0.034

These findings demonstrate that regression modeling can elucidate formula-specific dose-response patterns and provide a mathematical rationale for dose adjustment. However, the fitted models are formula-specific, reflecting differences in herbal composition and pharmacological action. Consequently, dose adjustment strategies derived from such models should be individualized rather than generalized. This study supports the development of tailored dose-adjustment protocols in clinical practice, based on the distinct dose-response characteristics of each TCM formula.

#### 2.1.5 Determination of the ‘dose adjustment range’

To establish a specific, quantifiable rule for dose adjustment, we analyzed data from a GQD trial featuring a well-defined, multi-dose gradient design. The proportion of patients achieving the predefined threshold of a ≥0.5 mmol/L reduction in fasting plasma glucose (FPG) at 4 weeks across dose groups is presented in Table 4. The 13 mg group showed the highest response rate (47.17%), which was superior to that of the medium-dose (9 mg) group (45.28%) and other dose groups. Therefore, for patients not meeting the FPG reduction target at 4 weeks, adjusting the dose from the initial medium dose (9 mg) to 13 mg—an absolute increase of 4 mg, corresponding to a relative increase of approximately 44.4%—was identified as the optimal choice supported by the data. This increment can be pragmatically summarized in clinical practice as “an increase of approximately half the standard dose,” providing a key, memorable, and actionable parameter for implementing the symptom-driven dose adjustment strategy.

**TABLE 4:** Least-squares means (LSMEANS) and 95% confidence intervals for the change in HbA1c (%) from baseline, adjusted for medication adherence (PPS).

Level/Comparison	Treatment level/Comparison	LSMean	95% CIL	95% CIU
Change in HbA1c (%) from baseline	Fixed-dose group	−0.03	−0.29	0.36
​	Symptom-driven dose adjustment group	−1.02	−1.32	−0.71
​	Fixed-dose group vs. Symptom-driven dose adjustment group (difference)	1.05	0.53	1.57

## 3 Prospective studies: prospective validation studies

### 3.1 Study design

To further validate the symptom-driven dose adjustment strategy proposed in the retrospective analysis, a randomized, parallel-group, multicenter clinical trial was conducted. The study included a 2-week run-in period followed by a 12-week treatment period. Number of cases: 30 in the study group and 30 in the control group.

#### 3.1.1 Inclusion criteria

Age 30–70 years; diagnosed with type 2 diabetes mellitus (T2DM); no regular use of glucose-lowering medication in the past 3 months; after 2 weeks of dietary control and exercise therapy, meeting either of the following criteria: (1) fasting plasma glucose (FPG) >7.0 mmol/L and <13.9 mmol/L with HbA1c ≥7.0%, or (2) 2-h postprandial glucose (2hPG) > 11.1 mmol/L with HbA1c ≥7.0%; signed informed consent; presenting with the traditional Chinese medicine syndrome pattern appropriate for treatment with GQD.

#### 3.1.2 Exclusion criteria

Pregnant or lactating women. Individuals allergic to Chinese herbal medicine components. Patients with severe complications involving the heart, liver, kidneys, brain, or other major organs, or those with other serious primary diseases. Individuals with psychiatric disorders. Patients who have experienced diabetic ketoacidosis, ketoacidosis, or severe infections within the past month. Patients whose primary condition is complications of diabetes mellitus. Blood lipid, blood pressure, or uric acid levels exceeding the upper limit of normal by 20%. Individuals who refuse to take Chinese herbal decoctions.

#### 3.1.3 Study groups and intervention protocol

Ninety-two patients with T2DM were enrolled and randomly assigned in a 1:1 ratio to two groups.

Fixed-dose group (control, n = 46): Received standardized GQD treatment.

Prescription composition: Puerariae Radix 24 g, Scutellariae Radix 9 g, Coptidis Rhizoma 9 g, Glycyrrhizae Radix 6 g.

Administration: Each daily dose was decocted to yield 300 mL of liquid, administered orally as 150 mL twice daily.

Dose regimen: The dose remained fixed throughout the 12-week treatment period.

Symptom-driven dose adjustment group (intervention, n = 46): Underwent a stepwise dose-adjustment regimen guided by fasting plasma glucose (FPG) levels.

Baseline treatment: Initial dose was identical to the control group (one dose per day).

Adjustment criteria:

FPG reduction ≥0.5 mmol/L: Maintain the current dose.

FPG reduction <0.5 mmol/L: Increase the dose by 50% (i.e., to 1.5 doses per day).

Adjustment schedule: A maximum of two dose adjustments were permitted, scheduled at Week 4 and Week 8.

- Standard Dietary and Exercise Methods
  - Dietary Control Methods: Follow the standards outlined in the Chinese Diabetes Prevention and Treatment Guidelines.
- Observation Indicators and Monitoring Points:
  - FPG and 2hPG (measured pre-treatment and every 2 weeks post-enrollment).
  - HbA1c, lipid profile, complete blood count, ALT, BUN, Cr (measured at 0 and 12 weeks post-enrollment).
  - Body weight, waist circumference, hip circumference, blood pressure, clinical symptoms, and changes in tongue and pulse (measured every 2 weeks).
  - Height (once before treatment).
  - Adverse events: Closely observe and document as they occur.

#### 3.1.4 Control of confounding factors

Dietary control was conducted according to the Chinese Guidelines for the Prevention and Treatment of Diabetes, and exercise therapy followed the same guidelines, ensuring that all patients maintained relatively stable dietary habits and exercise levels. The types and doses of lipid-lowering and antihypertensive medications remained unchanged throughout the study. No hypoglycemic medications other than those specified in the study protocol were added during the study period. Any other medications required for concomitant diseases were documented in detail.

#### 3.1.5 Data management

A dedicated database was established using EpiData. Data managers reviewed the case report forms, and any questions were addressed in writing by the investigators via a “Data Inquiry Form.” Data entry was performed by two independent operators working in duplicate; discrepancies were cross-checked item by item and corrected. Ten case report forms were randomly selected for manual comparison with the database. Logical checks were conducted in accordance with the clinical study protocol, and any questions were addressed again via the inquiry form. The verified database was locked at the data review meeting and handed over to the statistical analysts for use.

### 3.2 Statistical analysis

The study employed stratified analysis using the full analysis set (FAS), per-protocol set (PPS), and safety set (SS). Primary efficacy endpoints (changes in HbA1c, FPG, and 2hPG from baseline) were assessed for intergroup differences via analysis of covariance (ANCOVA), adjusting for baseline values and medication dosage as covariates. Least squares means (LSMeans) and their 95% confidence intervals were calculated. Intra-group efficacy was validated using paired t-tests. All hypothesis tests employed a significance threshold of α = 0.05, with statistical analyses performed using SAS software.

### 3.3 Research findings

To ensure adequate statistical power, the study initially planned for two groups of 30 participants each; and actually 46 participants were enrolled in each group, meeting the requirements for analysis. Dataset Disposition and Primary Outcomes: A total of 92 subjects were enrolled and randomized to the control group (conventional fixed-dose regimen, n = 46) or the study group (symptom-driven dose adjustment, n = 46). The full analysis set (FAS) included 45 subjects per group; the per-protocol set (PPS) comprised 35 controls and 39 study subjects; and the safety set (SS) included all 46 subjects in each group. Regarding primary outcomes: (a) HbA1c—In the FAS, the mean change from baseline was −0.62% (P < 0.0001) for the study group versus −0.26% (P = 0.0034) for controls; after adjusting for baseline, the least-squares mean difference was 0.34 (95% CI: 0.05 to 0.63, P = 0.0234), and after further adjusting for medication adherence it was 0.32 (95% CI: −0.03 to 0.68, P = 0.0746). In the PPS, the study group showed a mean change of −0.71% (P < 0.0001) versus −0.31% (P = 0.0068) for controls; after adjusting for baseline and adherence, the differences were 0.36 (95% CI: 0.04 to 0.69, P = 0.0279) and 1.05 (95%CI: 0.53 to 1.53, P = 0.0002), respectively (Tables 5–7). (b) FPG—In the FAS, mean changes were −0.83 mmol/L (P < 0.0001) in the study group and −0.42 mmol/L (P = 0.0643) in controls, with no statistically significant between-group difference after adjustment (P ≥ 0.0696). In the PPS, after adjusting for adherence, the difference was 1.19 mmol/L (95% CI: 0.20 to 2.17, P = 0.0186), favoring the study group (Table 8). (c) 2hPG—In the FAS, mean changes were −1.68 mmol/L (P < 0.0001) for the study group and −0.93 mmol/L (P = 0.0687) for controls; however, between-group differences remained non-significant regardless of adjustment (adjusted P = 0.1773 in FAS; P = 0.1839 in PPS).

**TABLE 5:** The proportion of patients with a decrease in FBG exceeding 0.5 mmol/L at 4 weeks across all dose groups (GQD).

Dose group (mg)	FPG reduction >0.5	FPG reduction <0.5
0	11 (20.37%)	43 (79.63%)
1	18 (33.96%)	35 (66.04%)
2	21 (39.62%)	32 (60.38%)
3	22 (40.00%)	33 (60.00%)
4	20 (37.74%)	33 (62.26%)
5	21 (39.62%)	32 (60.38%)
6	17 (32.08%)	36 (67.92%)
7	18 (33.96%)	35 (66.04%)
8	19 (35.85%)	34 (64.15%)
9	24 (45.28%)	29 (54.72%)
10	23 (43.40%)	30 (56.60%)
11	24 (45.28%)	29 (54.72%)
12	19 (35.85%)	34 (64.15%)
13	25 (47.17%)	28 (52.83%)
14	16 (30.19%)	37 (69.81%)
15	22 (43.14%)	29 (56.66%)
Total	320 (37.69%)	529 (62.31%)

**TABLE 6:** Changes in HbA1c (%) before and after treatment by group (PPS).

Measure	Fixed-dose group (n = 35)	Symptom-driven dose adjustment group (n = 39)
Baseline		
N (Missing)	35 (0)	39 (0)
Mean (SD)	7.97 (0.94)	8.06 (1.20)
Min, max	7,10.2	7,11.8
Md (Q3-Q1)	7.70 (1.50)	7.50 (1.30)
Week 12	​	​
N (Missing)	35 (0)	39 (0)
Mean (SD)	7.66 (0.92)	7.35 (0.94)
Min, max	6.2,10.1	5.8,11
Md (Q3-Q1)	7.40 (1.50)	7.20 (0.80)
Change (Week 12 - baseline)		
N (Missing)	35 (0)	39 (0)
Mean (SD)	−0.31 (0.63)	−0.71 (0.97)
Min, max	−2.2,1.8	−4.4,0.4
Md (Q3-Q1)	−0.30 (0.50)	−0.50 (1.00)
Paired t-test (P value)	−2.88 (0.0068)	−4.59 (<0.0001)

**TABLE 7:** Least-squares means (LSMEANS) and 95% confidence intervals for the change in FPG (mmol/L) from baseline, adjusted for medication adherence (PPS).

Level/Comparison	Treatment level/Comparison	LSMean	95% CIL	95% CIU
Change in HbA1c (%) from baseline	Fixed-dose group	−0.12	−0.74	0.50
​	Symptom-driven dose adjustment group	−1.31	−1.89	−0.74
​	Fixed-dose group vs. Symptom-driven dose adjustment group (difference)	1.19	0.20	2.17

**TABLE 8:** Least-squares means (LSMEANS) and 95% confidence intervals for the change in HbA1c (%) from baseline (PPS).

Level/Comparison	Treatment level/comparison	LSMean	95% CIL	95% CIU
Change in HbA1c (%) from baseline	Fixed-dose group	−0.33	−0.56	0.10
​	Symptom-driven dose adjustment group	−0.69	−0.91	−0.47
​	Fixed-dose group vs. Symptom-driven dose adjustment group (difference)	0.36	0.04	0.69

## 4 Discussion

### 4.1 Developing a standardized pathway for dosage adjustment of traditional Chinese medicine formulas: transitioning from empirical to evidence-based practice to decipher the “Black Box” of TCM dosing

This study provides the first evidence-based, data-driven framework for dose adjustment of traditional Chinese medicine (TCM) compound formulas, systematically addressing the long-standing clinical dilemma of when and by how much to adjust the dosage. In response to the complexity of herbal components, which precludes direct PK-PD modeling, we adopted an innovative strategy that circumvents mechanistic’black-box’constraints. By integrating the concept of a therapeutic window from conventional medicine and analyzing clinical data from 1,901 patients across 8 studies, we established quantifiable adjustment criteria: maintain the current dose if ΔFPG ≥0.5 mmol/L, and increase it by 50% if ΔFPG <0.5 mmol/L. Furthermore, a transferable research framework for dose optimization was developed, applicable especially to chronic disease management with TCM formulas, comprising four key steps: identification of critical nodes/indicators → quantification of decision thresholds → determination of adjustment magnitude → prospective validation.

By translating the traditional, experience-based principle of “symptom-driven dose adjustment” (e.g., ‘increase dose if ineffective’) into objective, data-informed decision rules—using FPG change and statistical models (ROC analysis, dose-response simulation)—this work bridges the gap between empirical practice and quantitative evidence. For instance, the rule “ΔFPG <0.5 mmol/L at week 4 → increase dose by 50%” offers a clear, operational guideline. This approach systematically demystifies the traditionally opaque process of dose individualization in TCM and supplies a replicable methodological framework to overcome its inherent limitations. It thereby lays a solid methodological foundation for developing standardized operating procedures (SOPs) for personalized TCM therapy and marks a paradigm shift from empirical judgment to data-driven, evidence-based decision-making in herbal medicine dosing.

#### 4.1.1 Data-driven core indicator and threshold determination

The key to the success of this study lies in the efficient and precise identification of core indicators and quantitative thresholds for guiding dose adjustments from clinical data. It should be noted that the process of selecting indicators and determining thresholds requires a large sample size to ensure statistical power and the generalizability of the conclusions. Therefore, we integrated data from all seven high-quality multicenter RCTs (1,809 patients) conducted in more than 10 Grade A tertiary hospitals nationwide (1,809 patients with 1,127 variables), thereby establishing a robust analytical foundation. Using SAS for standardized data processing and multivariate regression screening, the change in fasting plasma glucose (ΔFPG) was preliminarily identified as a potential sensitive marker for predicting the primary endpoint (ΔHbA1c). Subsequently, ROC curve analysis combined with the Youden index maximization principle was employed. Aligning the results with clinical relevance, ΔFPG at Week 4 was selected as the core indicator for dose titration. This finding significantly advances the critical decision window for TCM dose adjustment from the conventional 12-week endpoint to Week 4 of treatment, allowing for substantially earlier intervention. Finally, a clear, objective trigger threshold for dose adjustment was established: ΔFPG <0.5 mmol/L at Week 4. This measurable criterion serves as the foundational evidence required to transform dose adjustment from an empirical practice into a standardized, data-driven process. In recent years, biomarker-guided precision dosing strategies have been actively explored across various disease areas, including biomarker-guided antibiotic duration for sepsis, biomarker-based diagnosis and management of acute kidney injury, and galectin-3-targeted therapy after myocardial infarction (Dark et al., 2025; Yoon et al., 2022; Frangogiannis, 2023). In this study, we used ΔFPG as a trigger signal for dose adjustment, representing an initial attempt to apply this concept to traditional Chinese medicine (TCM) compound formulas. It should be noted, however, that the predictive accuracy of this indicator is limited, with a sensitivity of 49.02% and a specificity of 63.06%, and it can therefore only serve as an auxiliary reference for dose adjustment.

#### 4.1.2 Optimization of dose adjustment magnitude via dose-response simulation

To address the pivotal question of ‘how much to adjust,’ this study employed dose-response modeling to derive precise adjustment rules, which were subsequently validated in a prospective RCT. Using data from a GQD dose-ranging study (low/medium/high: 9 g, 13 g, 15 g), we simulated the relationship between the 4-week ΔFPG and the ultimate HbA1c improvement across doses. Simulations for patients with a suboptimal response (ΔFPG <0.5 mmol/L at week 4) revealed the following: maintaining the medium dose (9 g) resulted in an efficacy rate of approximately 45.28%; increasing the dose to 13 g (a 44.4% increase) raised the rate to 47.17%. In contrast, escalating directly to the high dose (15 g) yielded a slightly lower rate of 43.14%, suggesting that a simple’more is better’approach is not optimal and that an efficacious range exists. From these findings, a generalizable rule was formulated: for patients not meeting the target (ΔFPG < 0.5 mmol/L), the dose should be increased by 50% (i.e., a half-dose increment). This work directly resolves the clinical ambiguity surrounding the magnitude of dose adjustment.

It should be noted that Gegen Qinlian Decoction (GQD) consists of four herbal ingredients: Pueraria lobata, Scutellaria baicalensis, Coptis chinensis, and Glycyrrhiza uralensis. Modern pharmacological studies indicate that its hypoglycemic effects are associated with various active metabolites: puerarin, found in Pueraria, can lower blood glucose by improving insulin sensitivity and inhibiting glucose absorption and reabsorption (Yang et al., 2019); baicalein protects β-cells by scavenging free radicals, inhibiting protein kinase C, and activating α-glucosidase (Yingrui et al., 2022); berberine improves β-cell function by regulating glucose metabolism and activating the AMPK signaling pathway (Zhang et al., 2024); and licorice extract lowers blood glucose by increasing insulin receptor affinity and enhancing glucose utilization in peripheral tissues (Yang et al., 2020). The effectiveness of the “add half the dose” strategy in this study may be explained as follows: when fasting blood glucose does not decrease satisfactorily after 4 weeks, a moderate dose increase raises the plasma concentrations of these active metabolites, thereby enhancing their hypoglycemic effects. However, further increases (e.g., to 15 g/day) led to a decline in efficacy, which is speculated to be associated with increased gastrointestinal adverse reactions (e.g., mild diarrhea) caused by berberine or with target saturation. Although this study did not quantitatively analyze the chemical composition of each decoction batch and thus could not establish a precise dose–metabolite–effect relationship, the present dose-adjustment strategy remains pharmacologically rational based on existing literature and our dose-response simulations. Future studies should combine chemical fingerprinting and pharmacokinetic data to further validate the optimal range of this strategy.

#### 4.1.3 Prospective validation and paradigm shift

To validate the “symptom-driven dose adjustment” strategy (i.e., ΔFPG <0.5 mmol/L at week 4→increase dose by 50%), we conducted a prospective, randomized, controlled clinical trial (n = 92). The results demonstrated that the symptom-driven adjustment group achieved significantly greater reductions in the primary endpoint, HbA1c, compared to the fixed-dose group (FAS: 0.62% vs.–0.26%, P < 0.0001; PPS: 0.71% vs.–0.31%, P < 0.0001), with similar trends observed in other glycemic parameters. This prospective trial confirms both the scientific validity and the clinical effectiveness of the standardized, data-driven strategy derived from our framework, representing a paradigm shift in TCM dose individualization.

#### 4.1.4 Generalizability and transferability of the research approach

**FIGURE 1:**
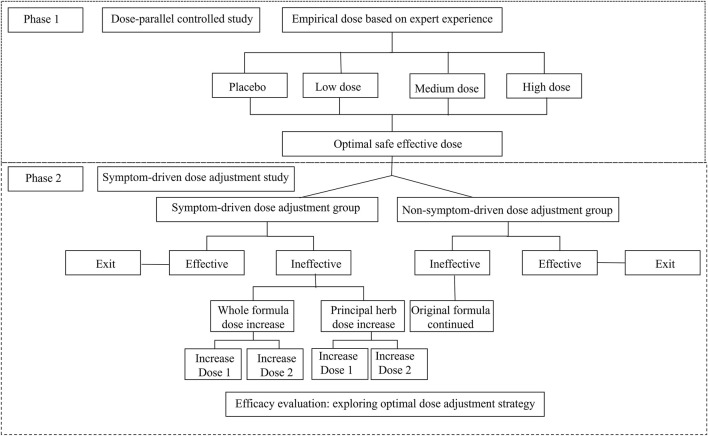
Clinical research pathway of the symptom-driven dose adjustment strategy.

The four-step framework established in this study-‘identify key nodes/indicators→quantify decision thresholds→determine adjustment magnitude→conduct prospective validation’—exhibits clear methodological generalizability. Although this paper uses T2DM and the formula GQD as examples, the core logic of this pathway—retrospective big data mining to identify early efficacy prediction indicators and thresholds, thereby deriving quantitative dosage adjustment rules—can be transferred to other Chinese herbal formulas, particularly in chronic disease management requiring long-term dose optimization (e.g., hypertension, chronic kidney disease). This establishes a replicable research paradigm for constructing data-driven, personalized TCM treatment plans across diverse disease domains. Figure 1 summarizes the complete decision-making pathway for this strategy, from initial treatment to dose adjustment, and can serve as a practical template for future studies.

### 4.2 Bridging traditional wisdom and modern methodology: toward adaptive design (SMART) integration

In recent years, the SMART design has demonstrated its advantages in the management of complex diseases in Western medicine, spanning multiple therapeutic areas including oncology (Kidwell et al., 2018; Khoury et al., 2024; Sikorskii et al., 2023), bone diseases (Mauck et al., 2025; Hisamune et al., 2024), obesity (Sherwood et al., 2022), and periodontal disease (Bahal et al., 2025). The dynamic decision-making logic of “assess-respond-adjust” embodied by the SMART design aligns closely with the principles of individualized treatment in traditional Chinese medicine. It has also begun to be applied in the field of TCM for chronic diseases (Pan et al., 2025). However, applying this approach to dose adjustment in TCM compound formulas requires addressing three key translational challenges: (1) How to determine appropriate decision-making time points for TCM? (2) How to select quantifiable response indicators that are readily accessible in clinical practice? (3) How to establish mathematical relationships for dose adjustment, making’dosing optimization’ operationally feasible. It is worth noting that this study did not initially adopt SMART design; however, through retrospective data mining and prospective validation, the constructed dose adjustment strategy aligns closely with the core elements of adaptive clinical trials. Specifically, based on analysis of 1,809 clinical cases, the study developed a dose adjustment protocol centered on 4-week FPG changes. This protocol embodies the intrinsic logic of SMART design in three aspects: (1) The predefined decision point (4-week assessment) aligns with the pharmacokinetic characteristics of TCM formulas; (2) Using continuous FPG change values as response indicators offers greater sensitivity than binary endpoints; (3) Establishing a quantitative dose adjustment rule (ΔFPG <0.5 mmol/L →increase dose by half). A prospective validation study (n = 92) demonstrated that this strategy significantly improved glycemic control compared to a fixed-dose regimen (p < 0.05). Although derived from retrospective analysis, these findings systematically address the three translational questions, providing crucial evidence for prospectively applying SMART design to TCM compound research. First, they validate the feasibility of adaptive adjustments based on early efficacy markers in compound drug therapy. Second, they demonstrate the efficacy of dose gradient adjustment (rather than drug switching) as an intervention option. Third, they offer methodological guidance for establishing standardized individualized treatment protocols for TCM compounds. Therefore, although this study is not a SMART trial, its results significantly enhance confidence in and feasibility for introducing SMART design in this field. Subsequent work can directly adopt the SMART framework to further optimize decision nodes and adjustment rules based on these findings.

### 4.3 Limitations

This study has the following major limitations. First, the area under the ROC curve (AUC) for ΔFPG in predicting ΔHbA1c was approximately 0.60–0.63, with a sensitivity of about 49% and a specificity of about 63%. This indicates modest predictive accuracy, suggesting that a single indicator provides limited information. We acknowledge that this indicator cannot serve as an independent diagnostic tool but should only be used as an ancillary trigger for clinical dose adjustment. Future studies should incorporate additional variables, such as 2-h postprandial blood glucose and early HbA1c changes, or employ machine learning models to improve predictive performance. Second, the retrospective data were derived from seven different studies, resulting in a certain degree of heterogeneity. The dose adjustment (increasing the dose by half) was based solely on data from Gegen Qinlian Decoction, and since this study used T2DM and this formula as examples, the generalizability of this strategy to other herbal formulas or chronic diseases requires individualized validation. Third, the prospective validation sample size was small (n = 92), and the phytochemical composition of the formulas was not standardized. Future studies should expand the sample size and incorporate chemical fingerprinting analysis.

## 5 Conclusion

This study achieved the “de-empiricization” and “standardization” of dosage adjustment for TCM formulas through multicenter randomized controlled clinical trial data. It not only resolved the core clinical challenges of “when to adjust dosage” and ‘by how much,’ but also propelled the shift in TCM personalized treatment from traditional experience to a modern evidence-based paradigm. This achievement provides a methodological foundation for precision medicine research on TCM formulas and holds broad clinical application prospects.

## Data Availability

The datasets presented in this article are not readily available because the dataset contains information that could compromise the privacy of research participants. Therefore, access is restricted to protect participant confidentiality and comply with the ethical approvals and informed consent agreements under which the original data were collected. Requests to access the datasets should be directed to FL Lfm565@sohu.com.
