# Supplementary material for: From empirical practice to precision medicine: developing a methodology for adjusting traditional Chinese medicine formula dosages based on eight multicenter clinical studies
Source: Front Pharmacol. 2026 Jun 29;17:1815285. doi: 10.3389/fphar.2026.1815285 (PMC13358450; doi:10.3389/fphar.2026.1815285)
Supplement: Supplementary file 1 [file Supplementaryfile1.docx]

Appendix

Area Under the ROC Curve for Predicting 12-Week HbA1c Decrease Based on Fasting Blood Glucose Decrease at Each Time Point

| **Timing** | **Area under the ROC curve** |
| --- | --- |
| 4 weeks | 0.601 |
| 6 weeks | 0.606 |
| 8 weeks | 0.630 |
| 10 weeks | 0.634 |
| 12 weeks | 0.633 |

Analysis of the Ability of 4-Week Fasting Blood Glucose Variation to Predict Treatment Efficacy

| **Decrease (mmol/L)** | **Effective** | **Invalid** | **Total** | **Test statistic** | **P-value** |
| --- | --- | --- | --- | --- | --- |
| Decrease by 0.1 |  |  |  | 5.37(Chi-square) | 0.0205 |
| Not achieved | 464( 41.21%) | 182( 48.02%) | 646( 42.92%) |  |  |
| Achieved | 662( 58.79%) | 197( 51.98%) | 859( 57.08%) |  |  |
| Total | 1126 | 379 | 1505 |  |  |
| Missing | 1 | 0 | 1 |  |  |
| Decrease by 0.2 |  |  |  | 9.40(Chi-square) | 0.0022 |
| Not achieved | 492( 43.69%) | 200( 52.77%) | 692( 45.98%) |  |  |
| Achieved | 634( 56.31%) | 179( 47.23%) | 813( 54.02%) |  |  |
| Total | 1126 | 379 | 1505 |  |  |
| Missing | 1 | 0 | 1 |  |  |
| Decrease by 0.3 |  |  |  | 13.46(Chi-square) | 0.0002 |
| Not achieved | 525( 46.63%) | 218( 57.52%) | 743( 49.37%) |  |  |
| Achieved | 601( 53.37%) | 161( 42.48%) | 762( 50.63%) |  |  |
| Total | 1126 | 379 | 1505 |  |  |
| Missing | 1 | 0 | 1 |  |  |
| Decrease by 0.4 |  |  |  | 15.92(Chi-square) | <.0001 |
| Not achieved | 547( 48.58%) | 229( 60.42%) | 776( 51.56%) |  |  |
| Achieved | 579( 51.42%) | 150( 39.58%) | 729( 48.44%) |  |  |
| Total | 1126 | 379 | 1505 |  |  |
| Missing | 1 | 0 | 1 |  |  |
| Decrease by 0.5 |  |  |  | 16.67(Chi-square) | <.0001 |
| Not achieved | 574( 50.98%) | 239( 63.06%) | 813( 54.02%) |  |  |
| Achieved | 552( 49.02%) | 140( 36.94%) | 692( 45.98%) |  |  |
| Total | 1126 | 379 | 1505 |  |  |
| Missing | 1 | 0 | 1 |  |  |
| Decrease by 0.6 |  |  |  | 14.69(Chi-square) | 0.0001 |
| Not achieved | 613( 54.44%) | 249( 65.70%) | 862( 57.28%) |  |  |
| Achieved | 513( 45.56%) | 130( 34.30%) | 643( 42.72%) |  |  |
| Total | 1126 | 379 | 1505 |  |  |
| Missing | 1 | 0 | 1 |  |  |
| Decrease by 0.7 |  |  |  | 13.91(Chi-square) | 0.0002 |
| Not achieved | 635( 56.39%) | 255( 67.28%) | 890( 59.14%) |  |  |
| Achieved | 491( 43.61%) | 124( 32.72%) | 615( 40.86%) |  |  |
| Total | 1126 | 379 | 1505 |  |  |
| Missing | 1 | 0 | 1 |  |  |
| Decrease by 0.8 |  |  |  | 9.25(Chi-square) | 0.0024 |
| Not achieved | 680( 60.39%) | 262( 69.13%) | 942( 62.59%) |  |  |
| Achieved | 446( 39.61%) | 117( 30.87%) | 563( 37.41%) |  |  |
| Total | 1126 | 379 | 1505 |  |  |
| Missing | 1 | 0 | 1 |  |  |
| Decrease by 0.9 |  |  |  | 10.83(Chi-square) | 0.0010 |
| Not achieved | 703( 62.43%) | 272( 71.77%) | 975( 64.78%) |  |  |
| Achieved | 423( 37.57%) | 107( 28.23%) | 530( 35.22%) |  |  |
| Total | 1126 | 379 | 1505 |  |  |
| Missing | 1 | 0 | 1 |  |  |
| Decrease by 1.0 |  |  |  | 11.31(Chi-square) | 0.0008 |
| Not achieved | 726( 64.48%) | 280( 73.88%) | 1006( 66.84%) |  |  |
| Achieved | 400( 35.52%) | 99( 26.12%) | 499( 33.16%) |  |  |
| Total | 1126 | 379 | 1505 |  |  |
| Missing | 1 | 0 | 1 |  |  |
| Decrease by 1.1 |  |  |  | 13.34(Chi-square) | 0.0003 |
| Not achieved | 765( 67.94%) | 295( 77.84%) | 1060( 70.43%) |  |  |
| Achieved | 361( 32.06%) | 84( 22.16%) | 445( 29.57%) |  |  |
| Total | 1126 | 379 | 1505 |  |  |
| Missing | 1 | 0 | 1 |  |  |

Mean and Standard Deviation of 4‑Week FBG Reduction in Each Dose Group of GQD

| Dose group(mg) | Mean reduction（mmol/L） | Standard deviation（mmol/L） |
| --- | --- | --- |
| 0 | -0.135 | 1.140 |
| 1 | 0.020 | 1.789 |
| 2 | 0.001 | 1.789 |
| 3 | 0.015 | 1.836 |
| 4 | -0.037 | 1.789 |
| 5 | -0.056 | 1.789 |
| 6 | -0.075 | 1.789 |
| 7 | -0.094 | 1.789 |
| 8 | -0.103 | 1.789 |
| 9 | -0.192 | 1.730 |
| 10 | -0.151 | 1.789 |
| 11 | -0.170 | 1.789 |
| 12 | -0.189 | 1.789 |
| 13 | -0.208 | 1.789 |
| 14 | -0.227 | 1.789 |
| 15 | -0.354 | 2.291 |

Changes in fasting blood glucose at each time point

| Time point | Fasting blood glucose (mmol/L) | | Change from baseline (mmol/L) | Paired t-test P value | |
| --- | --- | --- | --- | --- | --- |
| Baseline | 9.08±2.09 | — | | — | |
| Week 4 | 8.63±2.22 | -0.45±1.73 | | | <0.0001 |
| Week 6 | 8.49±2.16 | -0.59±1.74 | | <0.0001 | |
| Week 8 | 8.52±2.39 | -0.57±2.03 | | <0.0001 | |
| Week 10 | 8.32±2.30 | -0.76±1.97 | | <0.0001 | |
| Week 12 | 8.36±2.40 | -0.72±2.24 | | <0.0001 | |

## Changes in glycated hemoglobin (HbA1c)

| Time point | Mean ± SD | Median (Q1, Q3) | Range (Min, Max) | | P value |
| --- | --- | --- | --- | --- | --- |
| Baseline | 8.20±1.22 | 7.80 (7.30, 8.80) | 6.3, 16 | | — |
| Week 12 | 7.61±1.40 | 7.30 (6.60, 8.40) | 4, 16.5 | | — |
| Change(Week 12–Baseline) | -0.61±1.26 | -0.50 (-1.20, 0.00) | -8.5, 5 | <0.0001 | |

Summary of botanical ingredients, authentication, quality control, and safety of the included Chinese herbal formulas.

| Formula name | Botanical composition  (with dosage) | Botanical name  (with authority and family; pharmacopoeial drug name) | Chemical quality control information | Adverse events summary |
| --- | --- | --- | --- | --- |
| JTTZ（Jiangtang Tiaozhi Formula） | Aloe vera, Coptis chinensis, Anemarrhena asphodeloides, Monascus purpureus, Momordica charantia, Salvia miltiorrhiza, Schisandra chinensis, Zingiber officinale (dosages not reported) | Aloe vera (L.) Burm.f. [Asphodelaceae; Aloe]; Coptis chinensis Franch. [Ranunculaceae; Coptidis rhizoma]; Anemarrhena asphodeloides Bunge [Asparagaceae; Anemarrhenae rhizoma]; Monascus purpureus Went. [Monascaceae; Fermentum Rubrum]; Momordica charantia L. [Cucurbitaceae; Momordicae fructus]; Salvia miltiorrhiza Bunge [Lamiaceae; Salviae miltiorrhizae radix et rhizoma]; Schisandra chinensis (Turcz.) Baill. [Schisandraceae; Schisandrae chinensis fructus]; Zingiber officinale Roscoe [Zingiberaceae; Zingiberis rhizoma] | Not reported | 13 non‑serious adverse events (6.0%), 2 cases of transient ALT/AST elevation |
| Gegen Junyao (Gegen Qinlian Decoction(GQD)variant) | Low dose: Pueraria lobata 24 g, Scutellaria baicalensis 27 g, Coptis chinensis 27 g, Glycyrrhiza uralensis 18 g; Medium dose: Pueraria 72 g, Scutellaria 27 g, Coptis 27 g, Glycyrrhiza 18 g; High dose: Pueraria 120 g, Scutellaria 27 g, Coptis 27 g, Glycyrrhiza 18 g | Pueraria lobata (Willd.) Ohwi [Fabaceae; Puerariae lobatae radix]; Scutellaria baicalensis Georgi [Lamiaceae; Scutellariae radix]; Coptis chinensis Franch. [Ranunculaceae; Coptidis rhizoma]; Glycyrrhiza uralensis Fisch. ex DC. [Fabaceae; Glycyrrhizae radix et rhizoma] | HPLC fingerprinting; marker compounds: puerarin, baicalein, berberine | One patient in the low‑dose group withdrew consent and was excluded from safety analysis. Common adverse events: urinary tract infection, ECG abnormalities. |
| Huanglian Junyao (GQD variant) | Low dose: Pueraria 72 g, Scutellaria 27 g, Coptis 9 g, Glycyrrhiza 18 g; Medium dose: Pueraria 72 g, Scutellaria 27 g, Coptis 27 g, Glycyrrhiza 18 g; High dose: Pueraria 72 g, Scutellaria 27 g, Coptis 45 g, Glycyrrhiza 18 g |  |  | Common adverse events: positive urine protein, elevated urine leukocytes. |
| GQD (whole formula) | Low dose: Pueraria 24 g, Scutellaria 9 g, Coptis 9 g, Glycyrrhiza 6 g; Medium dose: Pueraria 72 g, Scutellaria 27 g, Coptis 27 g, Glycyrrhiza 18 g; High dose: Pueraria 120 g, Scutellaria 45 g, Coptis 45 g, Glycyrrhiza 30 g |  |  | No serious adverse events. |
| Qingre Jiangzhuo Formula | Coptis chinensis, Anemarrhena asphodeloides, Rheum palmatum, Prunus persica, etc. (specific proportions not reported) | Coptis chinensis Franch. [Ranunculaceae; Coptidis rhizoma]; Anemarrhena asphodeloides Bunge [Asparagaceae; Anemarrhenae rhizoma]; Rheum palmatum L. [Polygonaceae; Rhei radix et rhizoma]; Prunus persica (L.) Batsch [Rosaceae; Persicae semen] | Not reported | No serious adverse events. |
| Tang-Min-Ling-Wan（TM81）(Phase 3) | Proprietary Chinese medicine, 6 g per pill. Composition (publicly disclosed): Coptis chinensis, Rheum palmatum, Paeonia lactiflora, Bupleurum chinense, Trichosanthes kirilowii, etc. Exact proportions not disclosed. | Coptis chinensis Franch. [Ranunculaceae; Coptidis rhizoma]; Rheum palmatum L. [Polygonaceae; Rhei radix et rhizoma]; Paeonia lactiflora Pall. [Paeoniaceae; Paeoniae radix alba]; Bupleurum chinense DC. [Apiaceae; Bupleuri radix]; Trichosanthes kirilowii Maxim. [Cucurbitaceae; Trichosanthis radix] | Not reported | TM81 group: 24 mild adverse events (6.69%); among them, 2 cases of transient ALT elevation and 2 cases of AST elevation. |
| TM81(Phase 2) | Proprietary Chinese medicine, high dose 12 g/day, low dose 6 g/day (composition same as above) | Not reported (proprietary product) | Not reported | High‑dose group: 1 case of ALT elevation; low‑dose group: 1 case of diarrhea, 1 case of nausea. |
